# Oxygen Radicals Entrapped between MgO Nanocrystals: Formation, Spectroscopic Fingerprints, and Reactivity toward Water

**DOI:** 10.1021/acs.jpcc.3c06091

**Published:** 2023-11-22

**Authors:** Thomas Schwab, Eva Muchová, Korbinian Aicher, Thomas Berger, Milan Ončák, Oliver Diwald

**Affiliations:** † Department of Chemistry and Physics of Materials, Paris-Lodron University Salzburg, Jakob-Haringer-Straße 2a, Salzburg A-5020, Austria; ‡ Department of Physical Chemistry, University of Chemistry and Technology, Technická 5, Prague 166 28, Czech Republic; § Department of Ion Physics and Applied Physics, University of Innsbruck, Technikerstraße 25, Innsbruck A-6020, Austria

## Abstract

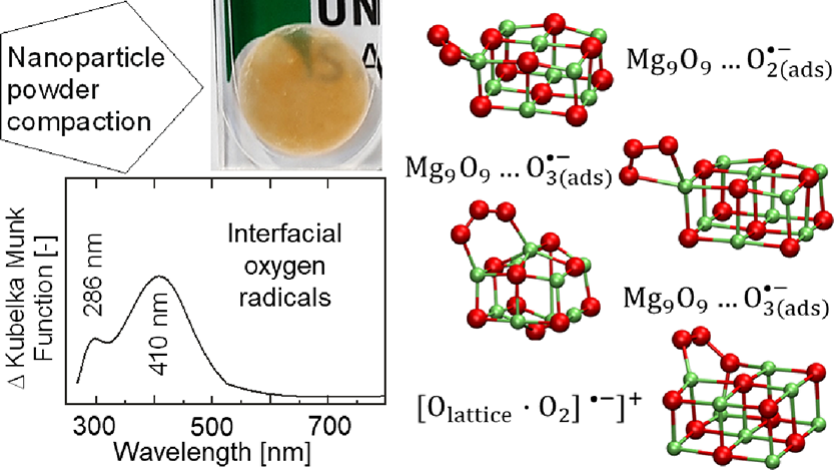

Compaction of dehydroxylated MgO nanocrystal powders produces adsorbed oxygen radicals with characteristic UV–vis spectroscopic fingerprints. Identical absorption bands arise upon UV excitation in an oxygen atmosphere but in the absence of uniaxial pressure. Photophysical calculations on MgO gas-phase clusters reveal that the observed optical transitions at 4.4 and 3.0 eV are consistent with adsorbed superoxide (O_2_^·–^) and ozonide (O_3_^·–^) species, respectively. The presence of these oxygen radicals is corroborated by electron paramagnetic resonance spectroscopy. Upon reaction with interfacial water, oxygen radicals convert into diamagnetic products with no absorptions in the UV–vis range. Since superoxide O_2_^·–^ and ozonide anions O_3_^·–^ play a key role in a variety of processes in heterogeneous catalysis, sensing, or as transient species in cold sintering, their UV–vis spectroscopic detection will enable in situ monitoring of transient oxygen radicals inside metal oxide powders.

## 1 Introduction

High surface area metal oxides host reactive interface structures at high concentrations. When excited with sufficient energy, related structures can undergo charge separation and charge transfer to adsorbates such as molecular oxygen.^1−3^ Analysis of such intergranular processes requires the isolation and identification of the reactive species involved and aims at mechanistic understanding for a variety of fields such as heterogeneous catalysis, tribochemistry, or sintering of nanomaterials.

Oxygen radicals fulfill important functions in a variety of biological, catalytic, and sensing processes.^4−9^ In biology, oxygen radicals are designated as reactive oxygen species (ROS), which are integral to cell signaling, apoptosis, and homeostasis.^10^ In sensing,^11^ oxygen radicals have been studied for their potential to detect trace amounts of carbon dioxide, hydrogen sulfide, and other gases. In metal oxide nanoparticle powders and high surface area materials for heterogeneous catalysis in general,^4,12^ oxygen radicals can control the rate of a catalyzed reaction and can increase the selectivity.

Electron paramagnetic resonance (EPR) spectroscopy is a powerful tool for the in-depth characterization of oxygen radicals and their interaction with solid surfaces. These paramagnetic species have, therefore, been exploited in model studies of metal oxide nanocrystal powders as powerful surface probes. At the surface of dehydroxylated MgO nanocrystals, paramagnetic lattice oxygen anions (O_lattice_^·–^) form upon UV-light-induced photoionization of low-coordinated surface sites. In addition, paramagnetic adsorbates such as superoxide (O_2_^·–^) and ozonide (O_3_^·–^) species have been identified when molecular oxygen was provided via the gas phase during UV excitation.^3,13,14^ Surprisingly, it was found very recently that the pressure-induced compaction of anhydrous MgO nanocrystal powders in the presence of residual oxygen gives rise to EPR spectra, which resemble the spectra obtained when exposing a compacted but adsorbate-free MgO nanocrystal ensemble to UV light in an oxygen atmosphere.^3^ Obviously, paramagnetic oxygen species may form^15^ as a consequence of charge separation at interface structures either (i) because of local forces between grains, which emerge upon uniaxial powder compaction,^3^ or (ii) upon sample exposure to photon energy.^13^

There exists a large body of experimental and theoretical evidence about the paramagnetic properties of radicals like superoxide ions, surface-trapped hole centers, or ozonide anions. In addition to electron paramagnetic resonance (EPR),^4^ analytical techniques like Raman spectroscopy^16,17^ or electrochemical sensing^18^ can provide valuable information about the structure, formation, and stability of adsorbed oxygen radicals and their transient role in surface chemistry.^15^ There is, however, a substantial lack of information about optical absorption property changes that can occur as a result of grain surface activation and consecutive oxygen radical formation.^3^ The origin of the optical transitions reported in ref (3) has so far remained unresolved, and related fundamental understanding, however, is required to further exploit the underlying effects for catalyst design and for new concepts of materials sintering.^19^ Theoretical modeling is one of the tools that can provide insight into optical transitions related to adsorbed oxygen radicals on the MgO surfaces.^15,20^ Computationally feasible cluster models help us to understand how the charged particles are created and how they interact with the surface.

In this spectroscopic study using UV–vis diffuse reflectance and electron paramagnetic resonance (EPR) combined with theoretical calculations, we provide the first-time evidence of characteristic optical absorption features related to oxygen radicals adsorbed at MgO nanocrystal surfaces. The measured spectra can now be assigned to calculated optical transitions of the paramagnetic oxygen species. We show that the reactivity of these radicals toward water vapor and conversion into diamagnetic product species can be tracked by time-dependent optical absorption measurements.

## 2 Methods

### 2.1 Experimental Section

#### 2.1.1 Particle Synthesis

MgO nanocubes were produced by chemical vapor synthesis (CVS) and flame spray pyrolysis (FSP). As outlined in a recent publication,^21^ both approaches in combination with subsequent sample annealing in alternating oxygen/vacuum atmospheres up to 1173 K give rise to comparable powder properties as well as particle size distribution and particle morphologies. In terms of optical absorption properties and paramagnetic properties, the material property changes that occur along powder compaction and UV excitation will be analyzed below in detail but are essentially identical for both types of materials.

Ad Chemical Vapor Synthesis (CVS): MgO nanocrystals obtained from chemical vapor synthesis (CVS) corresponded to the controlled combustion of Mg metal vapor in the presence of oxygen (O_2_ 5.0) under reduced pressure. The employed reactor system, the temperature program, nature, and flow rate of inert gas are provided in ref (21). The highly exothermic reaction of Mg vapor with oxygen leads to homogeneous nucleation and formation of MgO nanoparticles in the gas phase. Short residence times of resulting nuclei in the hot reaction zone (<2 ms), guaranteed by the continuous Ar flow (Ar 5.0) and pumping down to *p* = 50 ± 2 mbar, prevent undesired particle coarsening and coalescence.

Ad Flame Spray Pyrolysis (FSP): The self-constructed flame spray apparatus and its major parts are described in ref (21). The fuel-precursor mist was ignited by a concentrically arranged CH_4_/O_2_ (CH_4_ 4.5, 1.5 L/min, O_2_ 5.0, 2.0 L/min) combustion flame (supporting flame) surrounding the nozzle exit and converted into oxide monomers and volatile byproducts. Oxygen-rich environments for the synthesis of stoichiometric oxides and to avoid secondary byproducts were provided by an oxygen sheath flow (sheath gas, O_2_ 5.0, 5.0 L/min) guided through a sintered metal plate ring. Constant gas flow rates were adjusted via calibrated mass flow controllers (Bronkhorst EL-FLOW). A vacuum pump (Busch Seco SV 1040 C) ensures particle flow toward the particle collection unit that consists of a glass fiber filter located on a water-cooled filter holder (Hahnemühle, GF6, Ø 257 mm).

#### 2.1.2 Powder Annealing

Powder annealing was performed in dedicated fused silica cells attached to a high vacuum rack, which allows for pressures as low as *p* < 10^–5^ mbar and defined gas atmospheres. Sample heating up to 1123 K was described by a stepwise temperature increase of 100 K with heating rates of 5 K/min (room temperature up to 373 K) and 10 K/min (rest of the protocol). The next heating step was initiated as the pressure fell below *p* < 9 × 10^–6^ mbar. Admission of pure oxygen (*p*(O_2_) = 10 mbar) was performed at 1123 K and dwelled for 10 min. After subsequent evacuation to *p* < 9 × 10^–6^ mbar, the sample was heated to the final temperature (1173 K, *r* = 10 K/min), which was held for 60 min prior to cooling down to room temperature.^17,22^

#### 2.1.3 Ultraviolet**–**Visible (UV**–**Vis) Spectroscopy

UV–vis spectra in diffuse reflectance were acquired with a PerkinElmer Lambda 750 UV–vis–NIR spectrophotometer, equipped with an integrating sphere (*d* = 60 mm, Spectralon). Reflectance spectra were recorded with a spectral resolution of 2 nm by using a Spectralon diffuse reflectance white plate standard and were converted to absorption spectra by Kubelka–Munk transformation. The instrument uses a deuterium lamp for the energy range below 318 nm and a tungsten–halogen lamp for higher wavelengths. An optical high vacuum tight fused silica cell was used for spectroscopic investigation of compacted samples (*p* = 74 MPa) and allowed for optical measurements at pressures down to *p* < 10^–5^ mbar or in defined gas atmospheres.

#### 2.1.4 Electron Paramagnetic Resonance (EPR) Spectroscopy

X-band EPR measurements were performed on a Bruker EMXplus-10/12/P/L spectrometer equipped with an EMX^Plus^ standard cavity and using an NMR teslameter that allows for accurate determination of resonant field values. Green compact fragments (MgO) were transferred into a Suprasil quartz glass tube (*d* = 5 mm) and attached to the EPR system. This is connected to an appropriate high vacuum line with base pressures as low as *p* < 10^–5^ mbar and allows for in situ thermal treatment of the sample as well as the addition of pure gas atmospheres (*p*(O_2_) and *p*(H_2_O)). MgO powder compacts were investigated using a waveguide cryogen-free system (Oxford Instruments) to provide temperatures from 100 down to 10 K. For studies addressing the pressure-induced formation of paramagnetic species, the spectra of as-compacted samples (*p* = 74 MPa) were recorded at acquisition temperatures of 10, 100, and 298 K either under dynamic high vacuum conditions (*p* < 10^–5^ mbar) or in a pure oxygen atmosphere (*p*(O_2_) = 500 mbar). Additionally, spectrum acquisition under continuous pumping (*p* < 10^–5^ mbar) and at 10 K was performed on a powder compact, prior to and after contact with high-purity water vapor at room temperature (*p*(H_2_O) = 30 mbar, *t* = 90 min) to study the stability of emerging radical species.

Polychromatic sample excitation experiments on reannealed (*p* < 10^–5^ mbar, *T* = 1173 K) and EPR-silent powder compacts were carried out with a 300 W Xe-arc lamp equipped with a water filter to exclude IR contributions from the excitation light. Spectrum acquisition after UV excitation at room temperature and in a pure oxygen atmosphere (*p*(O_2_) = 100 mbar) through the aperture in the EPR cavity was performed under dynamic high vacuum conditions (*p* < 10^–5^ mbar) at 10 K. For EPR spectrum acquisition at a microwave frequency of 9.35 GHz, typical measurement parameters correspond to a field modulation frequency of 100 kHz, modulation amplitude of 0.1 mT, and a microwave power of 1 mW.

### 2.2 Computational Details

Gas-phase clusters were optimized at the PBE/aug-cc-pVDZ level of theory. More accurate calculations on the gas-phase O_2_^·–^ and O_3_^·–^ molecules were performed with the coupled cluster singles and doubles (CCSD) method. In the photophysical calculations, the CAM-B3LYP functional within the time-dependent density functional (TDDFT) formalism was applied, along with benchmarking using the equation of motion coupled cluster singles and doubles (EOM-CCSD) and multireference configuration interaction (MRCI) based on the complete active space self-consistent field (CASSCF) with various active spaces. The aug-cc-pVTZ basis set was used for O_2_^·–^ and O_3_^·–^, and the rest of the system was modeled using the smaller cc-pVDZ basis set. All transitions outside of the O_2_^·–^ or the O_3_^·–^ units were ignored. To this end, we calculated natural transition orbitals^23^ and considered only excitations by which initial and target orbitals were formed by at least 10% of O_2_^·–^ or O_3_^·–^ orbitals; the oscillator strength of the transitions was scaled by the respective percentage of the adsorbed ion orbital contributions. Only transitions with spin contamination below 40% were considered.

CASSCF/MRCI calculations were performed in Molpro v.2012.1,^24,25^ TDDFT calculations were performed in Gaussian 16,^26^ and EOM-CCSD calculations were performed in Q-Chem 6.0.^27,28^

## 3 Results and Discussion

### 3.1 Nanocrystal Powder Compaction, Color, and Changes in Optical Absorption

**Figure 1:**
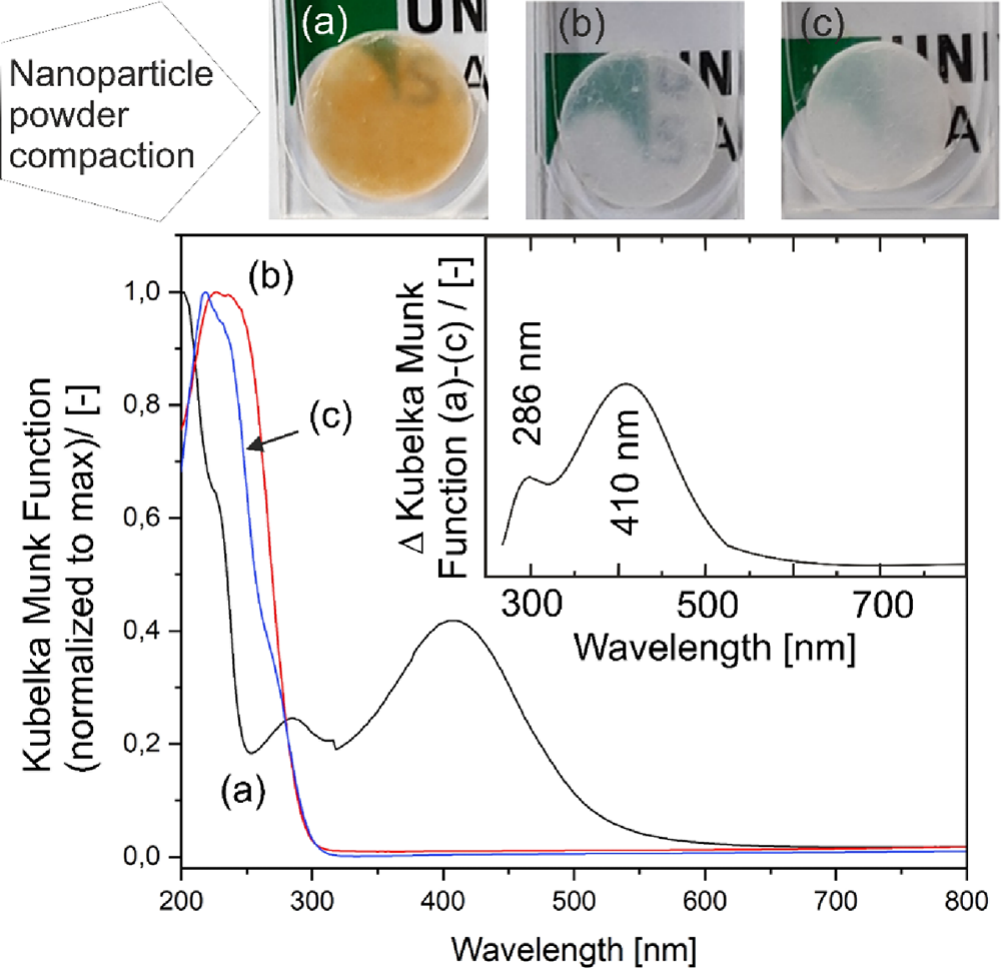
Digital photographs (upper panel) and diffuse reflectance spectra (lower panel) of MgO nanoparticle derived powder compacts (a) after compaction and spectroscopic measurement in the Ar atmosphere (or vacuum) ( after subsequent vacuum annealing to 673 K, and (c) after vacuum annealing to 1173 K with base pressures of *p* < 10^–5^ mbar.

Uniaxial pressing of MgO nanocube powders for compaction (*p* = 74 MPa, dwell time: 1 min) was performed with a hydraulic press (Atlas manual hydraulic press 15T, Specac) in an Ar gas atmosphere at room temperature. Prior to pressing, the annealed powder of defined mass (*m* = 150 ± 10 mg) was transferred within an Ar gas-filled glovebag from the fused silica cell (*p* < 10^–5^ mbar) into the cavity of a compaction tool (FTIR Pellet Dies, Specac). After pressing, the disk-shaped specimen was transferred (still within the glovebag) to a spectroscopic quartz glass cell for UV–vis diffuse reflectance measurements of the nanoparticle compact. Although powder compaction and sample transfer have been carried out in an Ar gas atmosphere, traces of residual water and oxygen are unavoidable. The digital photograph in the upper panel of Figure 1a clearly shows that the resulting nanoparticle compact has adopted a brownish color that originates from optical absorption bands with maxima at λ = 286 nm (4.3 eV) and 410 nm (3.0 eV) (Figure 1a, bottom panel, and Figure S3a). The latter absorption extends into the range of visible light (to λ = 600 nm) explaining the compact’s brownish-yellow color (Figure 1a, upper panel).

Vacuum annealing to 673 K annihilates all optical absorptions above λ = 300 nm, whereas further annealing to 1173 K, which typically enables perfect surface dehydroxylation, gives rise to a characteristic MgO specific absorption pattern with a band at 240 nm and a shoulder at 270 nm. These features are linked to the photoexcitation of dehydroxylated and low-coordinated ions in nanocube edges and corners, respectively.^29,30^

Significant changes in the optical absorption properties occur when a MgO nanocrystal compact, which was previously vacuum-annealed at 673 K to annihilate all optical absorptions above λ = 300 nm, is exposed in the presence of O_2_ to polychromatic UV light (Figure S1). Importantly, optical transitions at 4.1 and 3.0 eV (Figure S1b) are in very good agreement with the spectroscopic features observed after powder compaction (Figure S1a).

### 3.2 Electron Paramagnetic Resonance Evidence for Oxygen Radicals

**Figure 2:**
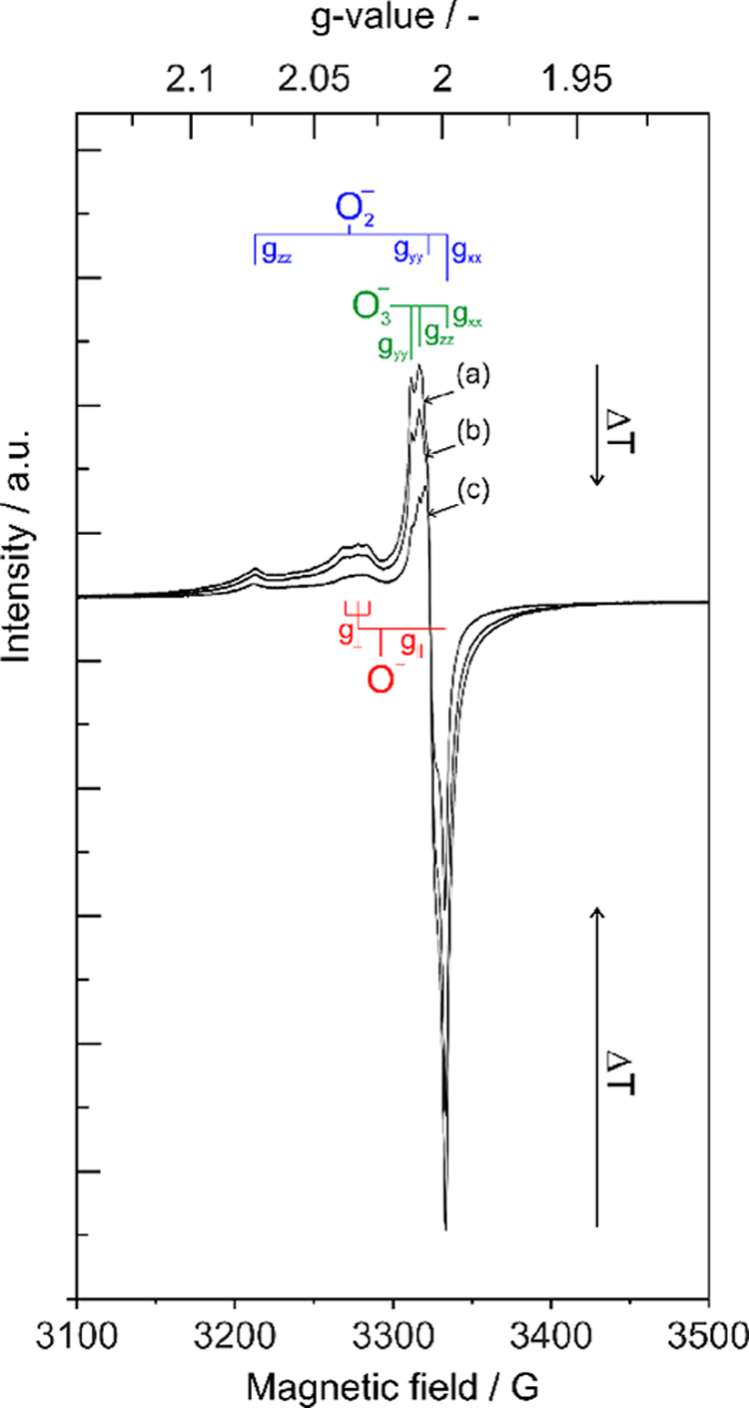
EPR spectra of MgO nanoparticle compacts acquired under dynamic vacuum and at three different temperatures: (a) 10 K, (b) 100 K, and (c) 298 K (O_2_ admission (500 mbar) produces only minor intensity changes and broadening of the individual signal components. Partial annihilation of the EPR features related to the O^–^ and O_3_^·–^ (see also Figure S2) is reversible with respect to the pressure of the O_2_ and points to the surface location of paramagnetic sites.).

EPR spectra were acquired on identically treated samples. For this purpose, the compact was broken into pieces that fit into the Suprasil sample tube with an inner diameter of 4 mm and stored under dynamic high vacuum conditions. The measured broad and complex EPR signal envelope (Figure 2) corresponds to the superimposition of signal contributions of at least three different types of oxygen radicals, which are O_lattice_^·–^ surface ions of the lattice (trapped hole centers), O_3_^·–^ (adsorbed ozonides), and O_2_^·–^ (superoxide anions).^3,14,15,21^ There are no significant variations of the signal shape observable when the acquisition temperature was changed from 10 K to room temperature. Corresponding paramagnetic oxygen species (Table 1) are stable at room temperature and continuous pumping, and the observed *T*-dependent intensity changes (Figure 2) are reversible.

**Table 1:** *g*-Parameters of Oxygen Radicals Observed (see Figure 2)

atmosphere	paramagnetic species	average *g*-values and shifts of EPR parameters at 10 K		
dynamic HV	trapped hole centers O^–^	*g*_⊥_ = 2.0323 ± 0.0006	*g*_∥_ = 1.9989 ± 0.0001	
	superoxide anions O_2_^·–^	*g*_*zz*_ = 2.07366 ± 0.0003	*g*_*yy*_ = 2.0046 ± 0,0001	*g*_*xx*_ = 1.9989 ± 0.0001
	ozonide anions O_3_^·–^	*g*_*yy*_ = 2.0115 ± 0.0009	*g*_*zz*_ = 2.0087 ± 0.0008	*g*_*xx*_ = 1.9989 ± 0.0001

in an O_2_ atmosphere	trapped hole centers O^–^			
	superoxide anions O_2_^·–^	*g*_*zz*_ = 2.07321 ± 0.0010	*g*_*yy*_ = 2.0045 ± 0.0002	*g*_*xx*_ = 1.9989 ± 0.0001
	ozonide anions O_3_^·–^			

The temperature-induced changes in the mean *g*-factor are in the range Δ*g* = 10^–4^ to 10^–3^, and corresponding shifts of the magnetic field positions Δ*B* ± 1.5 G are negligible and indicate that the paramagnetic oxygen species do not undergo any libration motions in this temperature range. Thus, oxygen adsorbs at spatially defined positions of the MgO grain surfaces, with which they strongly interact.

The EPR signal broadening observed clearly proves that a significant fraction of the underlying radicals is located at the outer grain surfaces where they undergo spin-exchange interaction with adsorbed molecular oxygen.

The mechanistic details of oxygen radical formation upon MgO nanoparticle powder compaction remain to be resolved. The experimental results clearly demonstrate that powder compaction gives rise to the local charge separation and interfacial charge transfer to adsorbates at the MgO surface (due to traces of residual oxygen contained in the sample environment upon pressing), which gives rise to essentially the same results as UV-light-induced excitation and charge transfer effects in the presence of oxygen (Figure S1):^3,31^
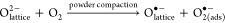
1

Compaction-induced charge separation and charge transfer lead to the formation of trapped electron and hole centers, i.e., adsorbed superoxide anions (O_2(ads)_^–^) and lattice oxygen anions (O_lattice_^–^), respectively. The latter may react further to give surface ozonide species ([O_lattice_·O_2_]^−^):
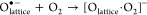
2

As a consequence of the reaction steps highlighted in eqs 1 and 2, at least three different paramagnetic species (i.e., O_lattice_^·–^, O_2(ads)_^·–^, and O_3(ads)_^·–^ ≡ [O_lattice_·O_2_]^−^ in eq 2) can be identified in the EPR spectra of the powder compacts (Figure 2). In the following, DFT calculations were performed to support the experimental findings and further characterize the spectroscopically observed oxygen radical species.

### 3.3 Computational Identification of Paramagnetic Oxygen Species at MgO Surfaces

**Figure 3:**
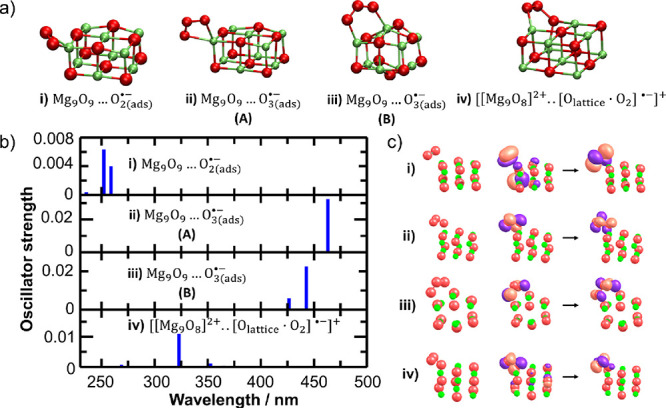
(a) Gas-phase models of O_2_^·–^ and O_3_^·–^ adsorbed on MgO clusters with either negative (first three models) or positive (fourth model) charge. (b) Position of electronic transitions in the respective models. Excitation energies were calculated at the TD-CAM-B3LYP level of theory, with the aug-cc-pVTZ basis set used for O_2_^·–^ or O_3_^·–^ and cc-pVDZ for the rest of the system (see the Methods section for details). (c) Character of bright electronic transitions in the O_2_^·–^ and the O_3_^·–^ adsorbed on MgO clusters in models **i**–**iv**. Natural transition orbitals are shown in panel (c).

Realistic models for processes described in eqs 1 and 2 should be neutral and should contain a sufficient surface area. However, it is difficult to create such models that would enable accurate modeling of the photophysics within a fully quantum chemistry approach, and here we use cluster models as the first approximation. We explored the photophysics of oxygen species on MgO employing models with adsorbed O_2_^·–^ and O_3_^·–^ (Figure 3a). We assume that the ozone molecule may be created from O_2_ during irradiation with sufficient energy (in the UV range, i.e., several eV) or through recombination on the surface; therefore, our models contain both adsorbed O_3_^·–^ and [O_lattice_·O_2_]^−^. Please note that our aim is not to investigate the detailed pathways of how the radicals are created on the surface but to provide photophysical data on the adsorbed oxygen radicals.

Models **i**–**iii** in Figure 3a are Mg_9_O_9_ clusters with adsorbed O_2_^·–^ and O_3_^·–^ anions. These models correspond to the charge transfer of a trapped electron (created by irradiation or compaction) in MgO to molecular oxygen or ozone and the subsequent adsorption on the surface. The overall charge after adsorption of O_2_^·–^ or O_3_^·–^ is negative. Model **iv** in Figure 3a corresponds to the presence of an anion vacancy center *F*_nc_^+^ on the MgO nanocubes. Here, we do not include the vacancy explicitly but assume that the Mg_9_O_9_ clusters bear a positive overall charge. This trick allows the investigation of the photophysics of the surface ozonide species [O_lattice_·O_2_]^−^.

First, we discuss the spectral features of the O_2_^·–^ anion in the gas phase. The ion has a bond length of 1.34 Å (as calculated at the CCSD/aug-cc-pVTZ level). Several excited states are localized in the 200–450 nm region; the most intense transitions are located at 250–280 nm (see Table S1). As can be inferred from Table S1, the calculations at the TDDFT level are in agreement with the more accurate EOM-EE-CCSD and MRCI data. The first valence transition of the ππ* character is positioned at 270 nm at the MRCI(9,12) level, and all other transitions below this wavelength correspond to transitions to diffuse virtual orbitals. Interestingly, the position of the ππ* transition matches well with the absorption peaks of O_2_^–^ measured at 245 and 255 nm in water and acetonitrile, respectively.^32^

When the O_2_^·–^ anion is adsorbed on MgO, it might be attached to the Mg^2+^ ion on the corner or on the edge. The bond length of the O_2_^·–^ bond in model **i** is slightly prolonged to 1.37 Å with respect to the gas-phase value; the Mg–O distances are 2.07 and 2.10 Å. From the photophysical perspective, the interaction with the MgO surface shifts the transitions to diffuse orbitals to higher energies and the ππ* transition (which is degenerate in the gas phase) splits into two transitions (see Figure 3b). Both transitions have the same character and partially involve orbitals of the MgO cluster. We note that the discussed excitations lie above the electron detachment energy, i.e., the reached excited states are metastable (see the Supporting Information for a detailed discussion). We thus attribute the experimentally observed broad absorption band at 286 nm to O_2_^·–^.

According to our calculations, the very broad peak centered at 410 nm observed in the experiment does not correspond to the O_2_^·–^ and must originate from another type of adsorbate. EPR experiments suggest that surface ozonides, O_3_^·–^, are present. At the (EOM)CCSD/aug-cc-pVTZ level, the optimized O–O bond length in the O_3_^·–^ is 1.34 Å and the OOO angle is 115°. The first bright excited state of O_3_^·–^ corresponds to the nπ* transition and can be found at ∼420 nm.

In our models, the ozonide molecule is adsorbed on the corner Mg^2+^ ion or forms a bridge structure (models **ii** and **iii** in Figure 3a). The bond lengths in the adsorbed O_3_^·–^ ion are slightly prolonged with respect to the gas-phase O_3_^·–^ to 1.35–1.38 Å; the Mg–O_3_^·–^ distances are 2.06 Å × 2.21 Å. The nπ* transitions are located at ∼440 and ∼465 nm, corresponding to 2.7 and 2.8 eV, and very close to the gas-phase transition, respectively (Figure 3b,c). We note that the nπ* transition lies below the electron detachment energy (see the Supporting Information). The excitation energies are also very close to the values observed for the ozonide radical in the aqueous solution.^33^

Alternatively, the ozonide structure might be formed through the interaction of O_2_ with the lattice oxygen in terms of eq 2 (model **iv** in Figure 3). The O–O distance of the adsorbed O_2_^·–^ is 1.31 Å, the distance of the O–O to the lattice oxygen is 1.46 Å, and the Mg–O distance is 2.08 Å. The structure lies somewhat between adsorbed O_2_^·–^ and O_3_^·–^. Accordingly, the calculated bright transition at 323 nm lies between the spectroscopic signals of the adsorbed anions. Being the mixture of the ππ* and nπ* transitions, the transition character can be interpreted either as the transition in the O_2_^·–^ for which the ground state is destabilized by the interaction with the lattice oxygen or as the transition in the O_3_^·–^ with a stabilized initial n state (see Figure 3b,c). The computational uncertainty enables us to draw only tentative conclusions and interpret the experimentally observed absorption at 410 nm in Figure 1 to be composed of absorption of adsorbed ozonide radicals, with O_3_^·–^ formed through the adsorption of O_2_^·–^ on the lattice oxygen playing a rather minor role. In the subsequent work, we will focus on the spectroscopic signals of the species adsorbed on the MgO surface in the periodic boundary conditions and their reactivity with other species.

### 3.4 Stability of Oxygen Radical Species in Aqueous Environments

**Figure 4:**
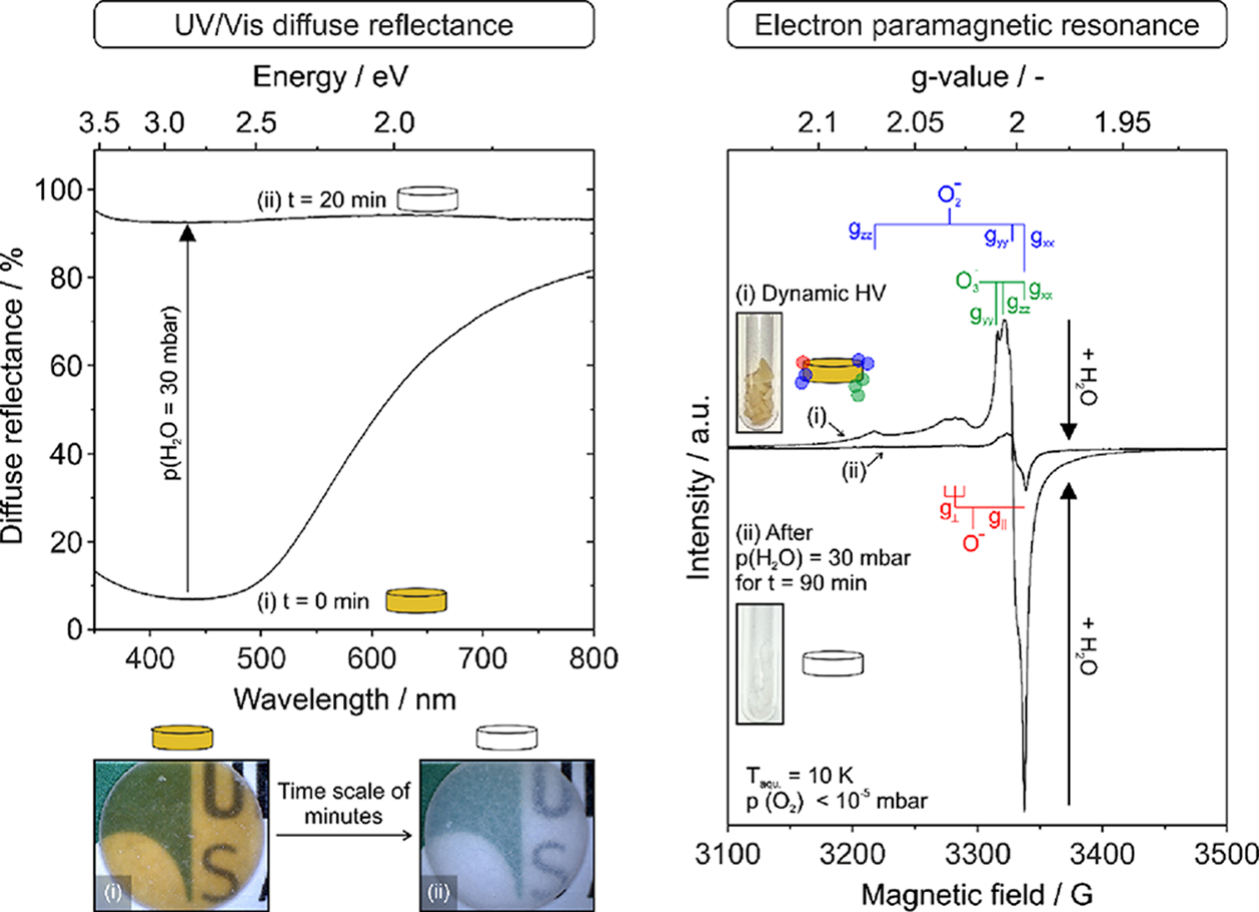
UV–vis diffuse reflectance (left panel) and EPR spectroscopic (right panel) evidence that reveal the reactivity of intergranular oxygen radicals toward water vapor. The spectra were acquired (i) prior to and (ii) after nanoparticle compact contact with water (*p*(H_2_O) = 30 mbar).

Translucency and optical absorption changes of the colored MgO nanoparticle compacts can be tracked with UV–vis spectroscopy both in the transmittance and in the diffuse reflectance mode in air and in the wavelength range between 300 and 800 nm (Figure 4, left panel, and Figure S3).

While storage of the MgO nanoparticle compact in anhydrous Ar and/or O_2_ atmosphere did not change the intensity of the brownish coloration, water vapor, which is an integral part of the ambient atmosphere, does. UV–vis measurements in diffuse reflectance (Figure 4) after exposing the MgO powder compact to water vapor (*p*(H_2_O) = 30 mbar) for 20 min reveal the extinction of the broad absorption signal between 350 and 700 nm on a time scale of minutes, as reflected by a fading of the brownish color (digital photographs (i) and (ii), bottom of Figure 4).

Despite differences in the pumping cross sections specific to the two spectroscopic setups and types of experiments, the EPR results in the right panel of Figure 4 were obtained under similar conditions as the UV–vis diffuse reflectance measurements. The EPR signal intensity fades away upon water adsorption (*t*(H_2_O) = 90 min, *p*(H_2_O) = 30 mbar) down to a level of 10% of its initial intensity. At the same time, the brown color of the compact inside the EPR tube bleaches out as indicated by the insets of the EPR figure (Figure 4, right panel), which supports the assumption that at least a fraction of the EPR active interface species do also contribute to the broad absorption in the visible range of light. Under ambient conditions and upon sample exposure to air, related absorption features fade away at a time scale of 1 day (Figure S3), whereas the H_2_O vapor-induced color bleaching occurs on the time scale of minutes (Figure 4, left panel).

While under the model conditions of computational chemistry superoxide anions seem to be stable in the presence of liquid water,^34^ there exists a complex interplay between such radicals in the surface-adsorbed form and different types of water molecules and hydroxyls that form upon ongoing surface hydration up to water film formation around the metal oxide grains.^35,36^ Thus, the detailed mechanism of O_2(ads)_^·–^ and O_3(ads)_^·–^ ion disproportionation and the conversion of these radicals into diamagnetic products at the hydrated metal oxide grain surfaces remain unresolved at present. However, the first steps may involve the protonation of superoxide anions.^37^
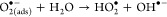
3followed by the transfer of a H atom originating either from another water molecule or from surface hydroxyls to the hydroperoxyl radical
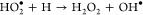
4to yield hydrogen peroxide and a short-lived OH· radical of enhanced chemical reactivity.^38^

Alternatively, or in parallel, reaction steps described along eqs 5–7 may occur
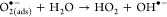
5
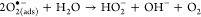
6
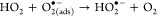
7to be followed by the annihilation of the hydroperoxyl anion (eq 8)^37^
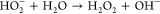
8

The results highlight that our methodical approach, which consists of investigating morphologically and compositionally well-defined MgO nanoparticle powders by a combination of spectroscopic techniques supported by DFT calculations, allows for the tracking and interpretation of currently poorly understood intergranular radical processes. From a technological point of view, triboemission-induced separation of surface charges and generation of surface radicals offer a novel opportunity region to initiate chemical reactions that can evolve in the confined space between the grains, which may lead to nanoparticle–polymer composites of superior homogeneity and density. Either initiated tribochemically or by UV excitation, such initial activation steps can also provide important mechanistic insights into cold sintering.

## 4 Conclusions

Uniaxial pressing of MgO nanocube powders at a pressure of *p* = 74 MPa produces characteristic spectroscopic property changes in the material, which are indicative of charge separation effects at the surfaces and interfaces of the nanograins. Identical spectroscopic features arise upon UV excitation in the oxygen atmosphere but in the absence of uniaxial pressure. By complementing the spectroscopic data from UV–vis reflectance and electron paramagnetic resonance experiments with results from photophysical calculations, a consistent picture of the underlying interfacial processes is gained. Upon pressure- or light-induced charge separation in an anhydrous gas atmosphere, molecular oxygen acts as a scavenger for both triboemitted and photogenerated surface electrons, respectively. At the same time, O_2_ stabilizes trapped electron–hole centers as surface O_lattice_^·–^ and O_3_^·–^ radicals, i.e., paramagnetic oxygen species, which convert into diamagnetic ones upon H_2_O adsorption. However, the oxygen radicals are characterized by limited chemical stability in the presence of interfacial water and convert into UV–vis silent diamagnetic products under ambient conditions.

This work shows that the chemical degradation of oxygen radicals can be followed by time-dependent optical absorption measurements. Spectroscopic observations on morphologically and compositionally well-defined materials under different environmental conditions together with the DFT calculation-based assignment of absorption features open an important methodical approach to the investigation of intergranular radical processes.

## Supporting Information Available

Complementary UV–vis diffuse reflectance spectra, computational details related to oxygen radical species in the gas phase and electron detachment processes, and Cartesian coordinates of all optimized structures (PDF)
