# Supplementary material for: Oxygen Radicals Entrapped between MgO Nanocrystals: Formation, Spectroscopic Fingerprints, and Reactivity toward Water
Source: J Phys Chem C Nanomater Interfaces. 2023 Nov 22;127(48):23332–9. doi: 10.1021/acs.jpcc.3c06091 (PMC10711787; doi:10.1021/acs.jpcc.3c06091)
Supplement: Supplementary file 1 — jp3c06091_si_001.pdf [file jp3c06091_si_001.pdf]

Supporting Information

Oxygen Radicals entrapped between MgO Nanocrystals: Formation, Spectroscopic Fingerprints and Reactivity towards Water

Thomas Schwab[1], Eva Muchová[2], Korbinian Aicher[1], Thomas Berger[1],

Milan Ončák[3*], and Oliver Diwald[1*]

[1]Department of Chemistry and Physics of Materials, Paris-Lodron University Salzburg, Jakob-Haringer-Straße 2a, A-5020 Salzburg, Austria

[2]Department of Physical Chemistry, University of Chemistry and Technology, Technická 5, Prague, 166 28 Prague, Czech Republic

[3]Department of Ion Physics and Applied Physics, University of Innsbruck, Technikerstraße 25, A-6020 Innsbruck, Austria

E-mail: milan.oncak@uibk.ac.at; oliver.diwald@sbg.ac.at

Table of Content

Experimental Section S2

Computations: Photochemistry of Oxygen Radical Species in the Gas Phase.....................S6

Computations: Discussion on the Electron Detachment Processes S7

Computations: Cartesian Coordinates of All Optimized Structures......................................S8

Experimental Section

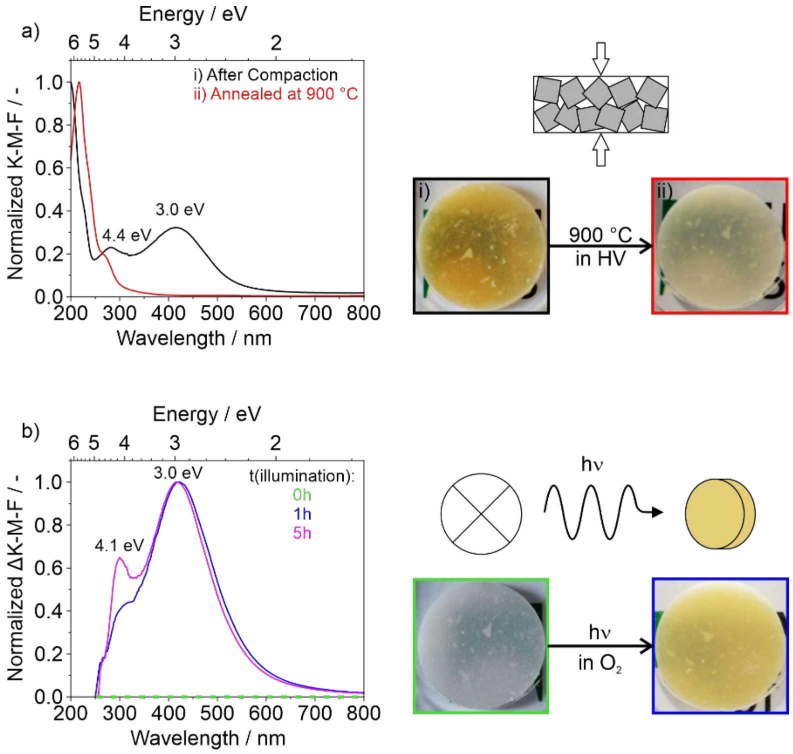

Figure S1: Comparison of compaction induced changes of the optical absorption properties of a MgO nanocrystal powder (a) with those produced by UV excitation of a compact (which was previously vacuum annealed at 673 K to annihilate all optical absorptions above λ = 300 nm) in O_2 atmosphere (b).

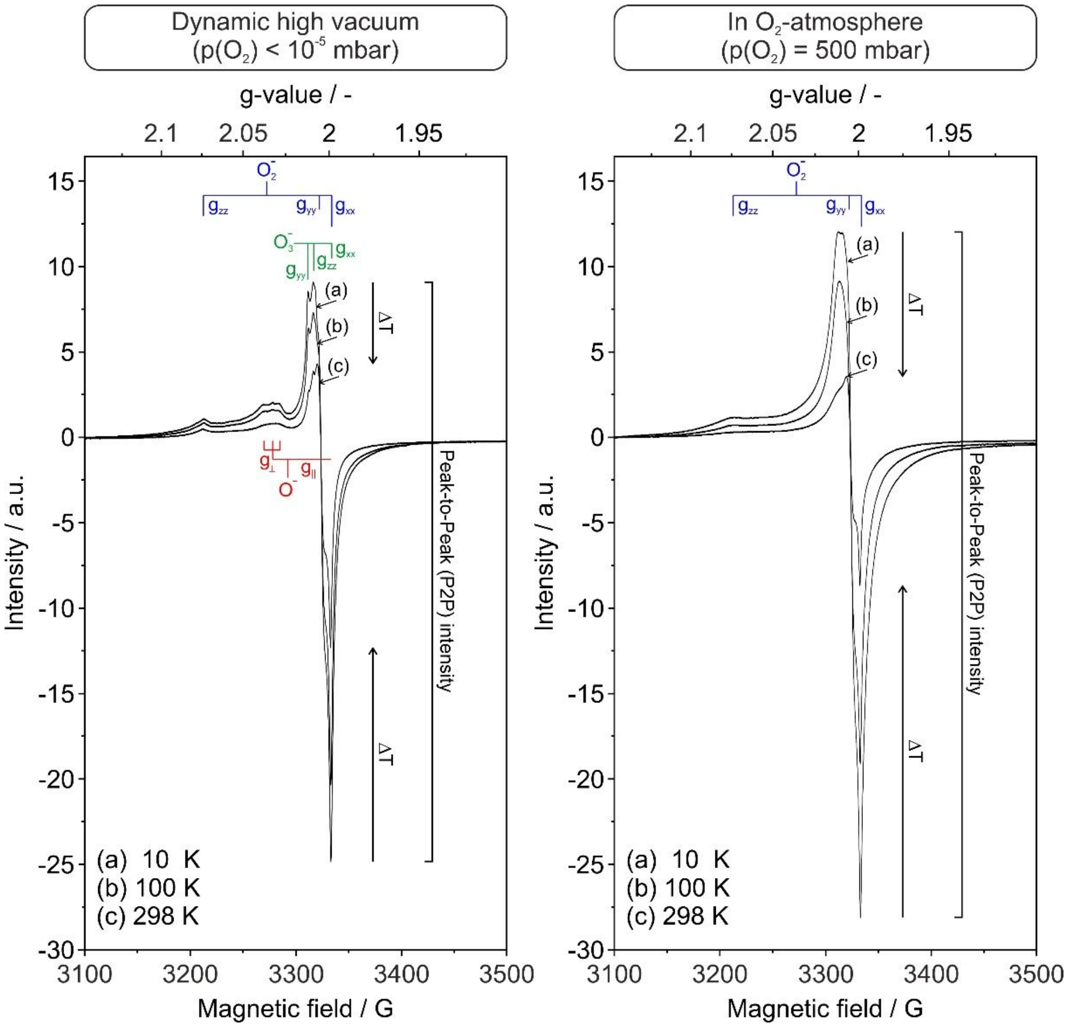

Figure S2: EPR spectra of MgO nanoparticle compacts acquired under dynamic vacuum conditions and at three different temperatures: a) 10K, b) 100K and c) 298K; (Admission of O_2 (500 mbar) broadens the individual signal components produces only minor intensity changes and annihilates the O^- related EPR features.

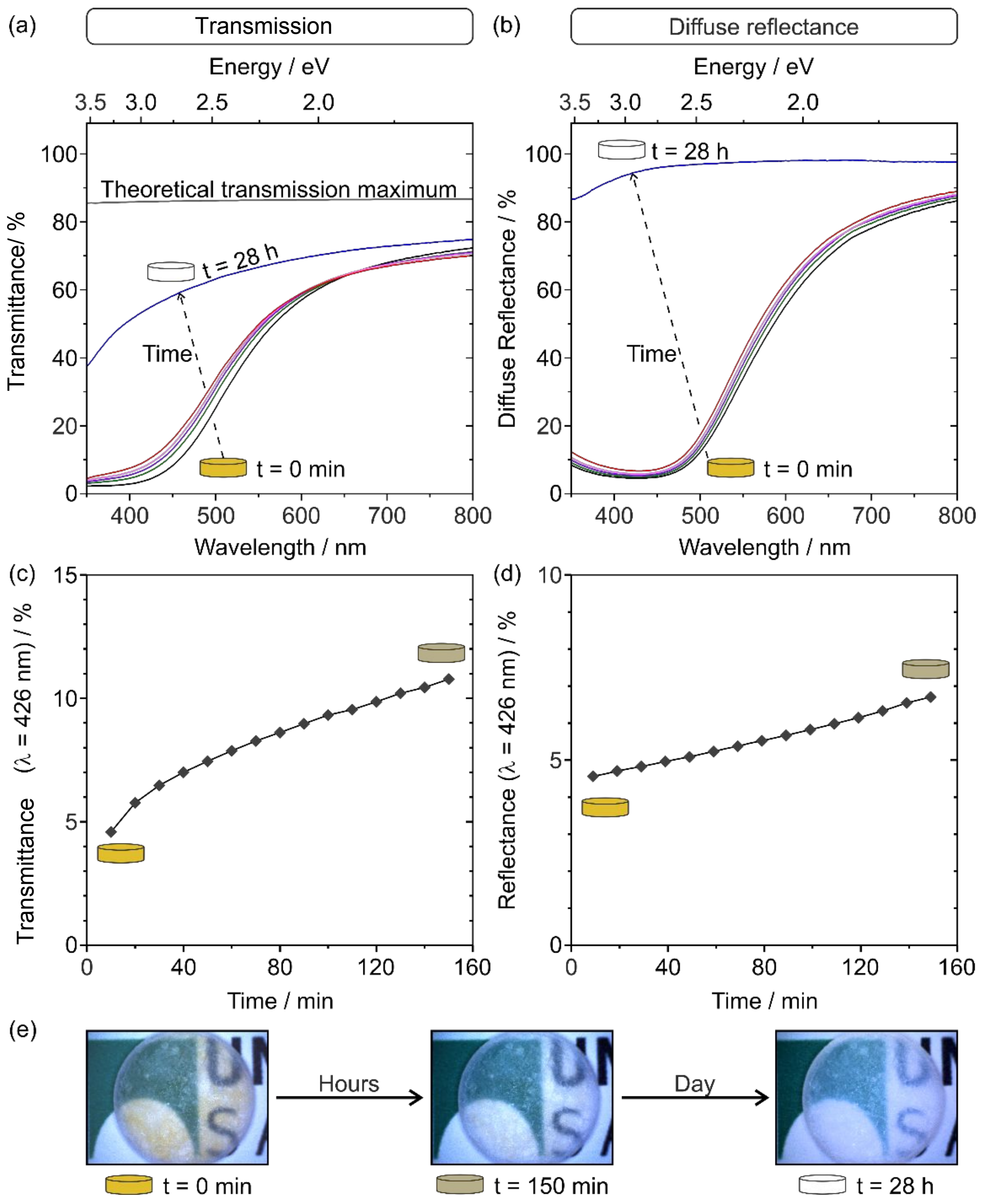

Figure S3: UV/Vis transmittance (left) and diffuse reflectance (right) spectroscopy data acquired on MgO powder compacts in air (top row a, b). Spectra acquisition was performed in defined time intervals of 10 min for integral measurement times of 2.5 h and after 28 h. The time evolution of the transmittance and diffuse reflectance data (middle row c, d) are plotted for λ = 426 nm, which corresponds to the absorption maximum. Digital photographs of respective powder compacts are provided in the bottom row (e).

Transmittance measurements performed on a translucent and brownish colored pellet (Figure S3a, top left) directly after pressing (t = 0 min) reveal a relatively high transmission value of roughly 70 % at λ = 800 nm. From 700 nm to smaller wavelengths, the transmission

strongly decreases with a minimum value around 426 nm. Storage of the pellet under ambient conditions leads to a continuous increase of the transmission signal at the time scale of hours (see time dependence of the signal at λ = 426 nm, Figure S3c). Within a period of 150 min the transmission signal increases from 4.5 % to 11 %. After 28 h of sample storage in ambient conditions the transmission increases over the entire wavelength range (between 40v70 %), but remains below the theoretical transmission maximum of transparent MgO.(Krell et al. 2009)

Complementary diffuse reflectance measurements (Figure S3b, top right) show a similar time dependence of the UV/Vis spectra. A strong decrease in diffuse reflectance from 700 nm to lower wavelength values and with a minimum at λ = 426 nm, which corresponds to the absorption maximum. The increase in reflectance (decrease in absorption) at 426 nm from 5 % up to 7 % at the time scale of 150 min (Figure S3d) is consistent with the optical transmittance measurements and reflects the fading of brownish coloration with time (compare Figure S3e). Within 28 h of sample storage in ambient atmosphere the pellet lost its brownish color and the reflectance curve, which lacks any characteristic absorption feature, is strongly shifted towards values of 80-95 %.

Computations: Photochemistry of Oxygen Radical Species in the Gas Phase

Table S1. Excitation wavelength λ (in nm) and oscillator strength *f* for bright transitions in $O_2^{\bullet -}$ in the gas phase (for the structure optimized at the CCSD/aug-cc-pVTZ level) calculated at various levels of theory. All methods employed the aug-cc-pVTZ basis set. EOM-EE-CCSD and TDDFT/CAM-B3LYP are not multireference methods and as such are not capable to describe the doubly degenerate ground state of $O_2^{\bullet -}$.

method	λ	f	λ	f	λ	f	λ	f	λ	f	λ	F
transition	4						3		2		1	
EOM-CCSD	228	0.0007	256	0.0700	265	0.0462	285	0.0104	350	0.0162	401	0.0080
MRCI(9,12)			255(2x)	0.0232			270(2x)	0.1217	286	0.0065	332	0.0073
MRCI(9,8)			266(2x)	0.0106			292(2x)	0.0436	308	0.0021	358	0.0019
TDDFT	231	0.0014	260	0.0856	269	0.0296	273	0.0110	365	0.0151	416	0.0087

Table S2. Excitation wavelength λ (in nm) for a bright transition and oscillator strength *f* of $O_3^{\bullet -}$ in the gas phase calculated using EOM-EE-CCSD and TDDFT/CAM-B3LYP. The aug-cc-pVTZ basis set was employed.

method	λ	f
EOM-CCSD	418	0.0751
TDDFT	445	0.0679

Computations: Discussion on the Electron Detachment Processes

An important process which must be addressed in the studied energy range is the electron detachment energy (EDE) of the $O_2^{\bullet-}$ adsorbed on the MgO surface. While the EDE of $O_2^{\bullet-}$ is in the gas phase 0.45 eV (Ervin et al. 2003), the MgO surface stabilizes the extra electron and shifts the EDE to higher energies. The calculated EDE are 4.5 eV for $O_2^{\bullet-}$ adsorbed on Mg_9O_9 in model **i**. This means that the neutral states formed upon electron detachment are more stable than the excited states. Thus, after the π-π* transition takes place, the energy contained in the system enables an electron ejection and formation of more stable ionized states. The excited state can be thus viewed as a resonance and its finite lifetime might contributes to the width in the absorption spectra.

The electron detachment energy of $O_3^{\bullet-}$ is 2.10 eV in the gas phase.(Arnold et al. 1994) For $O_3^{\bullet-}$ adsorbed on Mg_9O_9, the energy is predicted to significantly increase to 4.6 eV and 4.7 eV for models **ii** and **iii**, respectively. Thus, for $O_3^{\bullet-}$, the EDE lies above the excitation energy of the first bright nπ* transition and the considered excited state is not a resonance as in the case of $O_2^{\bullet-}$.

Computations: Cartesian Coordinates of All Optimized Structures along with the Electronic Energies in a.u. Including Zero-Point Correction

model i
E = -2627.386565
O -1.227061 -1.515027 -0.653339
Mg 0.089766 -0.010177 -1.019246
O 1.562563 1.524175 -1.409587
Mg 2.836916 0.062895 -1.423894
O 3.454919 0.081845 0.410356
Mg 2.009506 1.462948 0.750296
O 0.710067 0.010364 1.129091
Mg -0.777473 1.369072 1.385267
O 0.617200 2.857202 1.098061
Mg 0.132427 2.706901 -0.772469
O -1.304008 1.426467 -0.665071
Mg -2.678062 -0.064526 -0.148843
O -2.065866 -0.065891 1.782489
Mg -0.702608 -1.426625 1.386831
O 0.770737 -2.839235 1.106161
Mg 2.085096 -1.372780 0.754963
Mg 0.275387 -2.718143 -0.764134
O 1.640484 -1.462897 -1.406100
O -4.465457 -0.666756 -1.004861
O -4.600009 0.635406 -0.610358

model ii
E = -2702.497223
Mg 0.435330 -1.421486 1.451189
Mg -2.326540 -1.339927 0.718690
Mg 0.545869 1.374532 1.417747
Mg -2.213791 1.494051 0.679254
Mg -0.481041 -2.728764 -0.716191
Mg 2.485051 -0.096141 -0.027141
Mg -0.252181 -0.027443 -1.000138
Mg -2.981991 0.075289 -1.504480
Mg -0.267600 2.693742 -0.791097
O -1.046252 -2.818404 1.136139
O 1.800642 -0.072236 1.878834
O -0.948585 0.031157 1.128457
O -3.664054 0.127068 0.305803
O -0.817064 2.875561 1.058108
O 1.030172 -1.541095 -0.564978
O -1.805992 -1.464359 -1.423384
O 1.147096 1.396155 -0.616512
O -1.689043 1.520155 -1.463483
O 4.382769 1.022949 -0.157827
O 5.025798 -0.027338 -0.723220
O 4.169853 -1.085396 -0.899685

model iii
E = -2702.491779
O -1.065539 -1.468330 0.549399
Mg -0.841133 -0.303047 -1.166416
O 0.721400 1.091598 -1.942348
Mg 1.799755 -0.510845 -2.146457
O 3.241185 -0.090526 -0.909690
Mg 2.081268 1.463056 -0.259526
O 1.648215 0.472643 1.454237
Mg 0.028301 1.732914 1.549978
O 0.995127 3.039616 0.307018
Mg -0.254464 2.506791 -1.047651
O -1.419052 1.392483 -0.037048
Mg -2.197745 0.043699 1.189320
O -0.995119 0.464506 2.697483
Mg 0.267013 -0.932409 2.009309
O 1.570656 -2.437695 1.327148
Mg 2.704645 -0.998631 0.726216
Mg 0.320897 -2.609803 -0.152113
O 0.442982 -1.826295 -1.901750
O -2.912905 -0.537935 -1.777675
O -4.070124 -0.398916 -1.098480
O -4.019635 -0.288739 0.277718

model iv
E = -2627.020704
O 3.398262 -0.873668 -1.181369
O 3.800141 0.354423 -0.941316
O 2.863098 1.106791 -0.106552
O 0.624956 1.750094 1.344078
Mg -0.544821 0.035074 1.099774
O -2.198702 1.215163 0.776249
Mg -1.695418 1.138218 -1.351381
O -0.632833 2.799239 -1.095620
Mg 0.963484 1.759721 -0.746547
O 0.063619 -0.023376 -1.029657
Mg 1.464043 -1.402746 -0.612383
Mg 2.075297 0.531377 1.687571
Mg -1.251408 -1.729847 -1.151670
O 0.260398 -2.919036 -0.709248
Mg -0.093988 -2.701845 1.174996
O -1.710923 -1.639394 1.006251
Mg -1.026205 2.757921 0.797901
O 1.185835 -1.158957 1.462790
O -2.763098 -0.521308 -1.491564
Mg -3.151486 -0.447854 0.412378
